# Cardiovascular Disease Risk in Individuals Following Plant-Based Dietary Patterns Compared to Regular Meat-Eaters

**DOI:** 10.3390/nu16071063

**Published:** 2024-04-05

**Authors:** Grace Austin, Jessica J. A. Ferguson, Shaun Eslick, Christopher Oldmeadow, Lisa G. Wood, Manohar L. Garg

**Affiliations:** 1 School of Biomedical Sciences & Pharmacy, University of Newcastle, Newcastle, NSW 2308, Australiashaun.eslick@mq.edu.au (S.E.); lisa.wood@newcastle.edu.au (L.G.W.); 2 Hunter Medical Research Institute, New Lambton Heights, NSW 2305, Australia; 3 School of Health Sciences, University of Newcastle, Callaghan, NSW 2308, Australia; 4 Macquarie Medical School, Macquarie University, Macquarie Park, NSW 2109, Australia; 5 Clinical Research Design, Information Technology and Statistical Support Unit, Hunter Medical Research Institute, New Lambton Heights, NSW 2305, Australia

**Keywords:** plant-based diets, vegetarian, vegan, cardiovascular disease, CVD, veganism, pesco-vegetarian, semi-vegetarian, CVD, CVD risk, cross-sectional, cohort

## Abstract

Plant-based diets (PBDs) have been associated with a lower risk of cardiovascular disease (CVD). The aim was to investigate the predicted 5-year and 10-year risk of developing CVD in individuals following PBDs compared to regular meat-eating diets. This cross-sectional study included *n* = 240 middle-aged adults habitually consuming dietary patterns for ≥6 months: vegan, lacto-ovo vegetarian (LOV), pesco-vegetarian (PV), semi-vegetarian (SV) or regular meat-eater (RME) (*n* = 48 per group). Predicted 5-year and 10-year CVD risks were quantified using the Framingham Risk Equation and the Australian Absolute CVD risk calculator, respectively. Multivariable regression analysis was used to adjust for age, sex, smoking status, physical activity, alcohol use and BMI. Over three-quarters of the participants were women, mean age of 53.8 yrs. After adjustments for potential confounders, there was no difference in the predicted risk of CVD between regular-meat diets and PBDs, although crude analyses revealed that vegans had a lower 5-year and 10-year predicted risk of CVD compared to RMEs. SVs, PVs and LOVs had lower CVD risk scores, however, not significantly. Vegans had a favourable cardiometabolic risk profile including significantly lower serum lipid levels, fasting blood glucose and dietary fats and higher dietary fibre intake compared to RMEs. This was the first study to purposefully sample Australians habitually following PBDs. We found that PBDs do not independently influence the predicted risk of CVD, although PBDs tended to have lower risk and vegans had significantly lower cardiometabolic risk factors for CVD.

## 1. Introduction

Adopting a plant-based diet (PBD) has become increasingly popular with market research across the globe; around 12% of the Australian and UK population are vegetarians (meat free) or consuming small amounts of meat [1,2]. In the US, 39% report to be actively incorporating plant-based foods, with approximately 6% of the population reporting to follow a vegan or vegetarian dietary pattern [3]. Reasons for following a PBD are driven by growing research into the potential benefits, such as weight reduction [4,5], reduced risk of diabetes [6], concerns for animal welfare and ethics, environmental sustainability and the overall positive perception by the public [7,8]. The term PBD can be characterised by high intakes of plant foods and low intakes of animal flesh and/or animal-derived products [9].

Cardiovascular disease (CVD) remains the leading cause of death across the globe, representing almost 30% of all deaths [10]. Therefore, it is paramount that evidence around the nutritional aspects of PBDs is established to evaluate their impact on cardiovascular health and to shape nutrition education, guidelines, and public health programs. Previous literature suggests that PBDs may be beneficial for cardiovascular health, with a recent meta-analysis of 12 prospective cohort studies reporting an inverse association between risk of all-cause mortality and coronary heart disease (CHD) mortality [11,12]. ‘The 45 and Up’ study is the only Australian prospective cohort study comparing various PBDs to non-vegetarian dietary patterns and demonstrated no significant difference in all-cause and CVD mortality between the groups [13]. However, limitations of this study included unmatched groups, with under 2% of the sample following a PBD; vegans and vegetarians could not be distinguished; dietary pattern information was not collected with verified tools; and lastly, the study was a secondary analysis conducted over a decade ago (2006–2014). Evaluation of this study emphasised the need for an up-to-date, population-based study primarily designed to evaluate the potential effects of various PBDs on human health.

Research surrounding PBDs has primarily been conducted overseas in the US and Europe. Firstly, in 2001, a 10-year prospective cohort study, ‘EPIC-Oxford study’ (*n* = 48,188) demonstrated that fish eaters and vegetarians had 13% and 22% lower rates of ischaemic heart disease than meat-eaters, respectively [14]. Secondly, the Atherosclerosis Risk in Communities Study, a 29-year prospective cohort study including 12,168 individuals from the US, demonstrated that higher adherence to a healthy PBD index was associated with a 16%, 31–32% and 18–25% lower risk of CVD, CVD mortality and all-cause mortality, respectively. Dietary guidelines overseas have started to shift to incorporate plant-forward messaging and highlight the benefits of a vegetarian style dietary pattern [15]. However, current Australian Dietary Guidelines are lagging behind, with no specific recognition towards plant-forward dietary patterns [16]. Moreover, the literature does not encompass a standardised way of defining PBDs or traditional meat-eating diets and requires urgent refinement to effectively compare and evaluate differences in health outcomes. Definitions of PBDs previously implemented in an Australian cohort by Ferguson et al. [17], originally adapted from Mihrshahi et al. [13] and aligned with the World Health Organisation (WHO) [18], are used in the current study to aid a universal understanding of dietary characteristics associated with various PBDs.

The aim of this study was to investigate the predicted 5-year and 10-year risk of developing CVD among middle aged Australians habitually following various PBDs compared to regular meat-eaters (RMEs). Findings from this research will not only provide current evidence of plant-forward eating patterns of Australians, but also inform food and nutrition policy, population-based dietary guidelines, and the design of future longitudinal studies investigating PBDs.

## 2. Materials and Methods

### 2.1. Study Population

The PBD Study was a cross-sectional study conducted at the Nutraceuticals Research Program, School of Biomedical Sciences & Pharmacy, University of Newcastle, Callaghan NSW, Australia. Participants attended a one-timepoint data collection over the period of November 2021 to March 2023. Data were collected from consented enrolled participants after an overnight fast (~10–12 h) by the lead investigator. The research protocol describing the study has been published elsewhere [19]. Briefly, participants were deemed eligible if they were adults aged 30–75 years and following one of five defined dietary patterns for ≥6 months. They were excluded if they were pregnant or breast feeding; had made significant changes to their dietary pattern or physical activity levels in the past 6 months; and/or had a current, or history of, diagnosed CVD such as myocardial infarction, coronary insufficiency, angina, ischaemic stroke, transient ischaemic attack, haemorrhagic stroke, peripheral artery disease, heart failure or pacemaker implant. Individuals who were habitually consuming one of five dietary patterns (48 = per group) for ≥6 months were recruited into the following groups (Table 1): vegan (nil animal products), lacto-ovo vegetarian (LOV; nil meat, inclusive of eggs ± dairy), pesco-vegetarian (PV; nil meat, inclusive of seafood with/without dairy ± eggs), semi-vegetarian (SV; meat consumption ≤2 times per week) or regular meat-eaters (RMEs; meat consumption ≥7 times per week). Table 1 is a compressed version of the screening criteria published elsewhere [19] which was used to categorise participants into dietary patterns based on the average frequency at which animal-based foods were consumed per week [17,19]. An Accredited Practicing Dietitian (APD) conducted eligibility assessments over the phone and a second APD was consulted if discrepancies arose. Public notice board flyers, word of mouth and publicity generated by media outlets, e.g., newspaper articles, radio announcements and social media networks/groups detailing the PBDS were used to recruit individuals from the community, and individuals who participated in earlier studies at our research facility were also invited to participate. Written informed consent was returned to the investigators as a mandatory requirement for enrolment in the study and, upon enrolment, participants were de-identified and assigned a numeric identification code for data handling. This study was approved by the University of Newcastle’s Human Research Ethics Committee (HREC 2020-0195).

### 2.2. Study Regime

Measurements collected by the lead investigator during the appointments included blood pressure (three serial measurements using an average of the final two); diet histories; fasted blood samples via venepuncture; height (cm) and waist (cm) measured to the nearest 0.5; and weight (kg) measured to the nearest 0.1 units and used to calculate BMI (kg/m^2^). The questionnaire included medical history, demographic and ethnicity information and physical activity history. Participants confirmed the duration of their current dietary pattern via in-person interviews with an APD. The durations of their dietary patterns were strictly based on animal product exclusion (as per screening criteria). Biochemical analyses, including lipids (total cholesterol, LDL-cholesterol, HDL-cholesterol, triglycerides, total cholesterol-to-HDL-cholesterol ratio), fasting blood glucose (FBG) and liver function tests (LFTs), were measured by the commercial pathology service provider, NSW Health Pathology.

### 2.3. Dietary Assessment

The AES^®^ food frequency questionnaire (FFQ) was employed to measure the qualitative intake of food groups as per the Australian Guide to Healthy Eating [16,20]. The AES^®^ food frequency questionnaire (FFQ) is an online self-administered 120-question food frequency questionnaire (FFQ) which examines food and nutrient intake over the preceding 3–6 months and has been validated in Australian populations [21]. FFQs are an accurate means to asses the usual intake of various food items relating to a specific food category over a longer period of time [22]. The AES reported 6–8 options for the daily, weekly, or monthly intake of foods, ranging from “Never”, “less than 1 per month”, “1–3 per month”, “1 per week”, “2–6 per week”, “1 per week”, “2–3 per day and “4 or more per day”. The frequencies of foods consumed were converted to daily equivalents and the total reported intakes of all questions related to a specific food group were recorded.

Diet histories are a more accurate means to assess nutrient intake than FFQs [22]. Therefore, to collect total energy intake and quantitative nutrient intakes, including macronutrients and micronutrients, an APD conducted a comprehensive two-day diet history of participants’ usual eating habits. Detailed information of food brands and portion sizes at every eating occasion across the day were collected. Data were analysed using version 10 ‘FoodWorks’ (Xyris^®^, Brisbane, Australia, sourced online), which sources nutritional data of food items from ‘AusBrands’ and ‘AusFoods’ (2019). If food products were not in the database, they were manually added using the product’s nutrient information panel, inclusive of fortification. Micronutrient and macronutrient data were presented as mean average consumed (mg/g) per day from the two diet histories.

### 2.4. Cardiovascular Disease Risk Outcome Assessment

Five-year absolute CVD risk was determined using the Australian Absolute CVD Risk Calculator [23]. This was developed by the National Stroke Foundation on behalf of the National Vascular Disease Prevention Alliance (NVDPA), now part of the Australian Chronic Disease Prevention Alliance (ACDPA) [24]. The Framingham Risk Equation [25] was employed to calculate the 10-year risk of developing atherosclerotic CVDs and is a single multivariable risk function defined as coronary death, myocardial infarction, coronary insufficiency, angina, ischaemic stroke, transient ischaemic attack, haemorrhagic stroke, peripheral artery disease and heart failure. Variables that are required for the equations include (i) sex; (ii) age; (iii) total cholesterol; (iv) HDL-cholesterol; (v) systolic blood pressure (SBP); (vi) treatment status for hypertension; (vii) smoking status; and (viii) diabetes status. Data utilised for the CVD risk prediction models were collected from the medical history and demographic questionnaire. Diabetes status was defined as current diagnosis/treatment with oral anti-diabetic medications and smoking status as yes/no. The remaining lipid variables and total and HDL cholesterol levels were collected from fasted blood samples and SBP taken from an average of two repeated measures during study appointments.

### 2.5. Covariates and Mediators

The necessary covariates for evaluating CVD risk have been included in the Directed Acyclic Graph (DAG) (Supplementary Figure S1), including age, sex, alcohol use, smoking status and physical activity levels. As dietary pattern and weight status are interrelated, BMI was assessed as a mediator [26]. A self-administered medical history and demographic questionnaire collected family history of CVDs, prescribed or over-the-counter medication(s), habitual supplement use, habitual consumption of alcohol, ethnicity, marital and occupation status, level of education, smoking status, income, and alcohol use.

Alcohol intake was categorised into two variables, ‘consuming more than 4 standard drinks per day at least once per month’ (yes/no) and ‘Less than two days alcohol free per week’ (yes/no) as per the National Health and Medical Research Council’s alcohol guideline classifications for ‘risky drinking behaviour’ [27] and Dietitians Australia recommendations to ‘aim for two alcohol free days per week’ [28], respectively. Smoking status was categorised as ‘non-smoker’ and ‘current smoker’. Family history of CVD was defined as having at least one parent with a diagnosed CVD. The International Physical Activity Questionnaire (IPAQ, Long Version October 2002) is a self-administered and validated questionnaire used to assess habitual physical activity levels. This was interpreted as the metabolic equivalent of task minutes per week (MET/week) to measure the energy cost of physical activities [29]. Overweight and obese classifications were based on a BMI of ≥25 kg/m^2^ [30].

### 2.6. Statistical Analyses

As established from previous estimates of variance in 10-year CVD risk scores using the Framingham algorithm (mean = 13.3, standard deviation (SD) ± 5.77) [31], to elicit 80% power at a significance level of 0.05 to detect a 25% (Δ = 3.33) difference in CVD risk scores among at least one group following a PBD compared to regular meat-eaters, *n* = 240 participants (48 per dietary pattern) were required. Data were assessed for normality by inspecting histograms and quantile plots, and presented as means and SD or median and interquartile range (IQR), and categorical data summarised as frequency (*n*) and percentage (%). One-way ANOVA or Kruskal–Wallis tests were used to assess differences across dietary pattern groups for dietary intake and cardiometabolic, medical/demographic and biochemical parameters. Fisher’s exact tests were used to compare qualitative dietary intake data and other categorical data. The DAG displays dependencies between outcome (CVD risk), exposure (dietary patterns presented in Table 1) and confounding/mediating variables to determine the minimum set of variables necessary to control for confounding (Supplementary Figure S1) [32]. Since BMI was identified as a mediator and not a confounder, and PBDs being a categorical variable with five levels, we used seemingly unrelated regression models which adjusted for confounders in the presence of a mediator to estimate the crude and adjusted differences in CVD risk between dietary pattens [22,32]. *p*-values were adjusted for using the Benjamini–Hochberg method to control the false discovery rate to 5%. Statistical analyses were conducted using StataCorp. 2016 (Stata Statistical Software: Release 17 (College Station, TX, USA: StataCorp LP)).

### 2.7. Subgroup Analyses

The 5-year and 10-year risks of CVD were analysed in subcategories that were determined according to the frequency of meat and fish intake per week. Data were obtained from a subset of 24 questions referring to frequency of intake for red meat, chicken, and fish from the AES FFQ [20]. The ‘Meat and Fish’ group was defined as the total intake of all meats including mince, beef, pork, liver, chicken, processed meats and fish/other seafood. The ‘fish’ group was defined as the intake of fish/seafood only. Lastly, the ‘red meat’ group was defined as the intake of mince, beef, pork, liver and processed meats. The 5-year and 10-year risks of CVD in each dietary pattern were also analysed using the same regression model in the primary analysis separately within demographic subgroup characteristics, like subgroup analyses in the ‘EPIC Oxford study’ [14]. These included age (≤60 and >60 years), BMI (overweight/obese BMI of ≥25 kg/m or not overweight/obese BMI of <25 kg/m), duration of dietary pattern (≤10 or >10 years), sex (men or women), smoking status (yes and no) and treatment of chronic disease (yes and no).

## 3. Results

### 3.1. Characteristics of the Study Population

**Figure 1:**
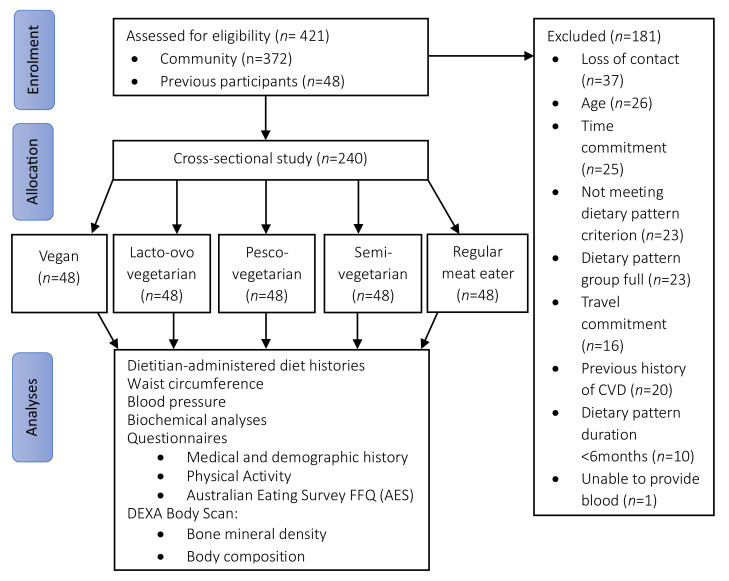
Flow diagram of participant recruitment, screening, and assessment. AES, Australian Eating Survey^®^; CVD, cardiovascular disease; DEXA, Dual Energy X-ray Absorptiometry; FFQ, food frequency questionnaire.

A total of 421 individuals were screened for eligibility and 240 participants were recruited into the study (Figure 1) across five dietary patterns (48 per group). Overall, more than three-quarters of the participants were women (78%) and aged 54 ± 10 years (range 31–74 years). Just under half (42%) were overweight or obese, with body weight of 69 ± 13 kg. The majority had a higher education (88%), approximately one-quarter were currently retired or not working (26%) and a small portion were current smokers (6%). Less than a quarter of the sample (21%) participated in ‘risky drinking behaviour’ as per the NHMRC guidelines by consuming more than four standard drinks at least once a month. One-quarter of the population were taking vitamin D supplements (25%) and a small portion consumed supplemental omega-3 polyunsaturated fats (n-3 PUFAs, 11%). The most-used medication was hormone replacement therapy (9%, in women only), followed by antihypertensive medications (6%), lipid lowering agents (3%) and oral glycaemic agents (1%).

### 3.2. Characteristics across Dietary Pattern Groups

Overall, when compared to participants following a regular meat diet, participants following a PBD tended to be younger, less likely to be overweight or obese, and participate in ‘risky drinking behaviour’; however, they had comparable rates of physical activity and distribution of sex (Table 2). Participant characteristics that were significantly different across groups included age, working status, duration of time following dietary pattern, alcohol use, n-3 PUFA use and eicosapentaenoic acid (EPA)/docosahexaenoic acid (DHA) supplement use. Vegans were younger than those following LOV, PV, SV and RME dietary patterns and reported lower rates of being retired/not working. RMEs had a longer mean duration of following dietary patterns of 32 yrs, whereas those adopting a PBD had a shorter mean duration: 7 yrs for vegans, 17 yrs for LOVs, 16 yrs for PVs, 11 yrs for SVs. Vegans were the only group that did not report less than two days alcohol free, followed by SV at 1%, which is comparably different to 6–7% in LOVs, PVs and RMEs. Eleven percent of RMEs reported use of n-3 PUFA and EPA/DHA supplements, compared to only 2–7% in those following PBDs. All other variables did not significantly differ when compared across dietary patterns, including race, smoking status, vitamin D supplement use, sex distribution, education status, physical activity, reported use of hormone replacement therapy, oral glycaemic agents, lipid-lowering agents, and antihypertensive medication.

### 3.3. Cardiometabolic Disease Risk Factors

Cardiometabolic variables, including current diagnoses and treatment of chronic health conditions, SBP, diastolic blood pressure (DBP), and biochemical analyses are presented in Table 3. Vegans had significantly lower fasting plasma and total cholesterol (TC) compared to all groups, and lower low-density lipoprotein-cholesterol (LDL-C) and non-high-density lipoprotein cholesterol (non-HDL-C) when compared to RMEs, PVs and LOVs, but not SVs. Comparing the RMEs to vegans, there was a significantly lower absolute mean difference of −0.9 mmol/L (−16%) for TC, −0.7 mmol/L (−20%) for LDL-C and −0.7 mmol/L (−18%) for non-HDL-C. FBG concentrations were significantly lower in vegans by 0.4 mmol/L (−8%) compared to RMEs. Additionally, individuals following a PBD were less likely to report having chronic health conditions such as hypertension and T2D; however, this did not reach significance. The remaining cardiometabolic measures were comparable across dietary patterns, including rates of diagnosed hyperlipidaemia, SBP, triglycerides (TG) and LFTs.

### 3.4. Quantitative Dietary Intake

The nutrient intakes of individuals following various dietary patterns are presented in Table 4. Total energy intake was comparable; however, most nutrients including protein as a percentage of energy intake (en%), starch, saturated fats, trans fats, PUFAs, cholesterol and dietary fibre significantly differed across groups. Protein (en%) intake was higher in RMEs compared to SV, PVs, LOVs and vegans by 3–5 en%, carbohydrate (en%) intake was 6–8% lower in those adhering to a regular meat diet compared to vegans, LOV and SV, and fat (en%) intake was comparable. Starch intake was higher in those following a vegan dietary pattern when compared to PVs and RMEs. Daily dietary fibre intake was lower in RMEs (33 g) when compared to all PBDs (42–58 g), with a 25 g/day difference between vegans and RMEs. Differences in fat intake between the two extreme dietary pattern groups demonstrated that vegans consumed 10 g less saturated fat, 0.7 g less trans-fat, and 237 mg less cholesterol, and on the other hand, 10 g more PUFA compared to RMEs. Monounsaturated fatty acids, sugar and alcohol were comparable across groups.

### 3.5. Qualitative Dietary Intake and Diet Quality

The daily intakes of the five food groups across dietary patterns are presented in Table 4. Vegans had a significantly higher fruit consumption compared to RMEs by an extra 1 serving/day. Consumption of meat/poultry/seafood/eggs/legumes/nuts was significantly higher for RMEs (2.7 serves/day) in comparison to PBDs (1.3–2 serves/day). Conversely, when subcategorising intake of legumes/beans/nuts, RMEs had a significantly lower intake compared to all groups, with a difference of 0.9 servings/day between vegans and RMEs. Vegans’ intake of dairy was nil and significantly lower compared to all other dietary patterns. Consumption of discretionary choices (including sugar-sweetened beverages) was higher in SVs and RMEs compared to vegans, LOVs and PVs. Non-significant trends revealed that vegans consumed an additional 1.3 servings of vegetables and 1.2 servings of grains on average per day compared to RMEs.

### 3.6. Dietary Patterns and Predicted 5-Year and 10-Year Risks of Cardiovascular Disease

After adjustment for potential confounders in a seemingly unrelated multivariable regression model, there were no significant differences in the predicted 5-year and 10-year risks of CVD between those following regular-meat diets and PBDs (Table 5). Sex, age, BMI and smoking status were significant variables within the model (Supplementary Figure S1). Crude analyses demonstrated that compared to RMEs, vegans had a significant inverse association in the 5-year predicted risk of CVD (−2.46, 95% CI −4.2, −0.7), equating to a −60% difference, and a significantly lower 10-year predicted risk of CVD (−3.20, 95% CI −5.4, −1.0) equating to a −44% difference. The other PBDs—SVs, PVs and LOVs—respectively followed the same trend, reporting a lower predicted risk of CVD in comparison to RMEs; however, the results were not statistically significant.

### 3.7. Association between CVD Risk and Frequency of Meat and Fish Intake

The predicted 5-year risk of developing CVD was significantly higher among individuals consuming red meat more than three times per week (*p* = 0.043) among RMEs and SVs; however, the effect was reduced after adjusting for confounders (estimate, 95% CI; see Supplementary Table S1). Intake of fish alone and all meats (red meat, chicken, and fish) three times a week or more showed inconclusive evidence of differences in the predicted 5- or 10-year CVD risk. The frequency of meat and fish intake was further subcategorised into three ordinal groups (≤2, >2 to <7, and ≥daily); however, outcomes were also inconclusive (Supplementary Table S2).

### 3.8. Subgroup Analyses

Exploration by demographic subgroups after adjustments demonstrated a significantly lower predicted 10-year risk of CVD in vegans only (*p* = 0.042) when compared to RMEs in individuals following a dietary pattern for ≤10 years, although lower scores were observed in the LOV and SV dietary pattern groups. Both 5- and 10-year CVD risk did not significantly differ between dietary pattern groups when stratified by other demographic subgroups: age (≤60 years, ≥60 years), overweight or obese BMI (yes, no), sex (male, female), smoking status (yes, no), treatment of chronic disease (yes, no) (Supplementary Table S3).

## 4. Discussion

This cross-sectional study of middle-aged Australians demonstrated that there were no significant differences in the predicted 5-year and 10-year risk of CVD between those following regular-meat diets and PBDs after adjustments for potential confounders, although crude analyses revealed that vegans had a significantly lower predicted risk of CVD and cardiometabolic risk factors for CVD compared to RMEs. SVs, PVs and LOVs had lower CVD risk scores; however, not significantly.

Previous findings demonstrate that PBDs are associated with a significantly reduced risk of CVD incidence [33,34]. A recent meta-analysis [34] reported significant reductions in the risk of CVD incidence, total stroke, and ischemic heart disease (IHD) in vegetarians (including vegans) compared to non-vegetarians. Several meta-analyses of prospective cohort studies demonstrate that vegetarian (including vegan) dietary patterns are associated with lower CVD, CHD and IHD mortality, although outcomes for all-cause mortality and stroke were inconclusive [12,35,36,37]. None of the published studies cohesively defined various types of PBDs, often including both vegans and laco-ovo vegetarians as ‘vegetarians’ and neglective to identify other PBDs including PVs and SVs. The current study used a specified screening criteria and qualified personnel to precisely categorise dietary patterns and demonstrated a lower risk of CVD among vegans compared to RMEs, although in the crude model only. This may indicate that vegans among the ‘vegetarians’ identified in previous literature may play a key role in driving the decreased risk of CVD incidence. Larger prospective longitudinal studies among Australians are warranted to substantiate these trends.

Confounding variables including sex, smoking status, age and BMI were significant factors for CVD risk prediction within this sample. Sex and age had the greatest influence on predicted CVD risk, with women and younger participants reporting lower scores. Both men and women in this sample had similar mean ages. Results from the Framingham Heart Study support sex differences in CVD outcomes, reporting women to have lower incidence at all ages except above 85 years old [38]. It is well known that smoking increases all-cause mortality and development of CVD and has been shown to double the 10-year risk of fatal events when comparing smokers to non-smokers [39]. In the present study, only 6% of the population were smokers, yet when compared to non-smokers, they had a 20% higher predicted 10-year risk of CVD. BMI had the smallest significant influence on CVD risk scores, and the rates of overweight and obesity were comparable across dietary patterns. Although the significant confounders in this study are well-known modulators of CVD risk, similar studies investigating PBDs and CVD clinical endpoints have reported that age, sex, smoking status and BMI did not significantly influence CVD risk [9,14]. This may suggest that the use of equations to predict CVD risk may be particularly sensitive to adjustments in comparison to actual clinical endpoints.

Vegans in this study had nutritional intakes favourable for heart health, including lower intakes of saturated fats, trans fats, cholesterol, and higher intakes of PUFA and dietary fibre. A systematic review and meta-analysis of 22 cohort studies found that total dietary fibre intake was inversely associated with CVD risk [40]. These outcomes align with the present study, as vegans (58 g/day) had significantly higher fibre intake by 25 g/day compared to the RME (33 g/day), which equated to more than double the recommended daily intake of 25–30 g/day [41] and suggested a daily target of 28–38 g for chronic disease prevention [20].

Pooled results from twelve prospective cohort studies showed trans fats, but not saturated fats, were associated with increased all-cause mortality, total CHD incidence, and CHD mortality, [42]. A meta-analysis of fifteen RCTs suggested that reducing dietary saturated fat reduced combined cardiovascular events by 21% [43]. A large prospective RCT supports these findings, showing that saturated fat intake was associated with higher risk of CVD and dietary intake of n-3 PUFAs to be inversely associated with all-cause mortality [44]. PUFAs, specifically n-3 PUFAs, are well known to protect against CVD through mechanisms such as lowering TG and LDL-C, improving endothelial function, acting as an antiatherogenic agent and reducing systemic inflammation [45,46]. These studies corroborate findings in the present study, as RMEs had the highest saturated fat intake, lowest PUFA intakes and highest mean predicted CVD risk. Conversely, vegans had the lowest saturated fat intake, highest PUFA intake and lowest mean predicted CVD risk.

Dietary fat profiles are further supported by serum lipid levels. Non-HDL-C, TC and LDL-C concentrations were significantly higher in the RMEs compared to vegans. Meta-analysis of twenty RCTs showed that consuming a vegetarian (including vegan) dietary pattern was associated with a 0.4 mmol/L reduction in LDL-C compared to RMEs [47]. Another meta-analysis of 30 RCTs illustrated that the vegetarian (including vegan) dietary pattern is associated with reduced TC (−0.34 mmol/L) and LDL-C (−0.30 mmol/L) compared with RMEs [48]. These findings support those observed in the current study, whereby in comparison to RMEs, vegans had significantly lower TC (−0.9 mmol/L) and LDL-C (−0.7 mmol/L). The same meta-analysis showed that SBP did not significantly change in the vegetarians (including vegans), analogous to the present study, suggesting that PBDs may not play a predominant role in directly moderating the risk/management of hypertension. FBG levels were significantly lower by 0.4 mmol/L in vegans compared to RMEs, aligning with a recent meta-analyses of RCTs reporting a 0.4 mmol/L reduction in FBG in vegetarians (including vegans) [49]. This study strengthens the developing recognition of PBDs as an effective tool in the prevention and management of type 2 diabetes and elevated serum lipid levels, which are key risk factors for CVD [4,50].

This study explored frequency of meat and fish intake and associated risk of CVD, which has been previously highlighted as a priority focus area for future PBD studies [9,14]. Those consuming red meat >three times/week had a significantly higher predicted 5-year risk of CVD, although results were non-significant after adjusting for confounders, suggestive of the potential interplay of other lifestyle factors. Higher intakes of meat, particularly red and processed meats, are well known to be associated with increased of CVD risk and all-cause mortality [51], and have been reported to be associated with significantly higher odds of diabetes in Australian women [6]. Outcomes from the present study support current guidelines from The National Heart Foundation of Australia for limiting unprocessed red meat intake to 1–3 meals/week to help reduce CVD risk [52].

This study included cohesively defined PBDs, utilised validated dietary assessment tools, examined and statistically accounted for an array of relevant confounders and provided up-to-date population-based evidence on the health parameters of individuals following various PBDs. Observational studies contributing towards much of the scientific knowledge base around PBD among Australians are from secondary analyses and neglect to provide comprehensive descriptions of various PBDs. The present study used definitions of PBDs we developed for an Australian cohort [6,17], adapted from Mihrshahi et al. [13] and aligned with WHO definitions [18] to create a cohesive and standardised understanding of dietary characteristics associated with various PBDs. This study was not completely free of limitations. Although adequately powered, the sample size of 240 participants was modest, which reduced the ability to adjust for a multitude of confounders. However, overadjustment was not an issue, as results were non-significant after adjustment of the initial set of necessary confounders. Being a cross-sectional study, CVD clinical endpoints associated with dietary patterns were not assessed; therefore, causality cannot be determined. The FFQ was self-reported data, which means it may be subject to recall bias; however, the tool implemented in this study has been validated in Australian populations [21]. We acknowledge selection bias as individuals volunteering to participate in the study may be from a pool of participants who might be better educated, of higher socioeconomic status or health-motivated [53]. Considering these limitations, this may act as a pilot study for larger prospective cohort studies investigating health outcomes associated with PBDs. Future studies should follow a consistent method of categorising well-known PBDs to ensure that the outcomes are true to the characteristics of each dietary pattern and translational to clinical practice.

## 5. Conclusions

In conclusion, this was the first study to purposefully sample Australians habitually following PBDs, presenting novel population-based up-to-date evidence on their potential influence on CVD risk. We found that PBDs do not independently influence the predicted risk of CVD, although PBDs tended to have lower risk, and vegans had significantly lower cardiometabolic risk factors for CVD. Larger population-based longitudinal studies primarily investigating the development of CVD in the context of cohesively defined PBDs are warranted.

## Figures and Tables

**Table 1:** Classification of dietary pattern groups by the number of time(s) foods are consumed on average per week, used for screening eligibility.

Times per Week Consumed	Vegan	Lacto-Ovo Vegetarian	Pesco-Vegetarian	Semi-Vegetarian	Regular Meat-Eater
Red meats	0	0	0	0 or <2	0 or ≥7
White meat/poultry	0	0	0	0 or <2	0 or ≥7
Processed/cured meats	0	0	0	0 or <2	0 or ≥7
Seafood/fish	0	0	≥1	0 or <2	0 or ≥7
Total of above categories	0	0	≥1	1 to ≤2	≥7
Usual eating habits:					
Animal-based dairy	0	Yes	n/a	n/a	n/a
Eggs	0	Yes	n/a	n/a	n/a

This table of the defining characteristics of dietary patterns is a compressed version of the previously published protocol [19] implemented previously [17]. ‘n/a’ denotes characteristics that were not relevant and thus not used for categorising participants into respective dietary patterns.

**Table 2:** Characteristics of participants across different dietary pattern groups.

	Total Sample (*n* = 240)	Vegan (*n* = 48)	Lacto-Ovo Vegetarian (*n* = 48)	Pesco-Vegetarian (*n* = 48)	Semi-Vegetarian (*n* = 48)	Regular Meat-Eater (*n* = 48)	*p*
Women	186 (77.5%)	34 (70.0%)	36 (75.0%)	39 (81.3%)	40 (83.3%)	37 (77.1%)	0.625
Age (yrs)	53.8 ± 10.3	47.8 ± 10.0 ^a^	53.7 ± 10.0 ^b^	55.8 ± 11.0 ^b^	55.2 ± 8.7 ^b^	56.5 ± 9.7 ^b^	<0.001
Ethnicity							
Oceanian	110 (45.8%)	23 (47.9%)	20 (41.7%)	21 (43.8%)	19 (39.6%)	27 (56.3%)	0.514
European ^1^	66 (27.5%)	15 (31.3%)	14 (29.2%)	10 (20.8%)	14 (29.2%)	13 (27.1%)	0.809
Other ^1^	64 (25.7%)	10 (20.9%)	14 (29.1%)	17 (35.4%)	15 (31.3%)	8 (16.7%)	0.238
Height (cm)	167.8 ± 9.1	170.1 ± 9.5	168.4 ± 10.2	167.2 ± 7.8	165.3 ± 9.7	168 ± 7.7	0.120
Weight (kg)	69.1 ± 13.3	70.4 ± 13.5	69.9 ± 13.4	67.6 ± 11.4	66.5 ± 14.4	71.25 ± 13.5	0.358
Overweight or obese	100 (41.7%)	17 (35.4%)	23 (48.0%)	20 (41.7%)	16 (33.3%)	24 (50.0%)	0.375
Higher education	42 (88.3%)	42 (87.5%)	41 (85.4%)	44 (91.7%)	41 (85.4%)	44 (91.7%)	0.767
Retired/not working	62 (25.8%)	4 (8.3%)	11 (22.9%)	16 (33.3%)	17 (35.4%)	14 (29.2%)	0.010
Physical Activity (MET)	5424 ± 5243	5775 ± 4036	6984 ± 8783	4909 ± 4101	4393 ± 3534	5060 ± 3600	0.143
Current Smoker	15 (6.3%)	5 (10.4%)	3 (6.3%)	1 (2.1%)	3 (6.3%)	3 (6.3%)	0.617
Family History of CVD	111 (46.3%)	19 (39.6%)	23 (47.9%)	27 (56.3%)	21 (43.8%)	21 (43.8%)	0.894
Dietary pattern duration (yrs)	16.5 ± 1.2	6.8 ± 7.7 ^a^	16.7 ± 14.1 ^b^	15.6 ± 14.7 ^a,b^	11.2 ± 13.8 ^a,b^	32.1 ± 24.7 ^c^	<0.001
Alcohol intake ^2^							
>4 drinks per month ^2^	50 (20.8%)	8 (16.7%)	11 (22.9%)	12 (25.0%)	10 (20.8%)	9 (18.8%)	0.917
<2 alcohol free days/wk	20 (8.3%)	0	6 (12.5%)	6 (12.5%)	1 (2.1%)	7 (14.6%)	0.005
Medication and Supplement use (%) ^3^							
HRT ^4^	21 (8.8)	2 (4.2)	5 (10.4)	5 (10.4)	4 (8.3)	5 (10.4)	0.724
OHA	2 (0.8)	0	0	0	1 (2.1)	1 (2.1)	1.000
Antihypertensive	15 (6.3)	1 (2.1)	3 (6.3)	6 (12.5)	1 (2.1)	4 (8.3)	0.199
Lipid lowering agent	8 (3.3)	0	1 (2.1)	2 (4.2)	3 (6.3)	2 (4.2)	0.653
N-3 PUFA ^5^	27 (11.3)	3 (6.3)	7 (14.5)	4 (8.3)	2 (4.2)	11 (22.9)	0.035
EPA/DHA	24 (10.0)	2 (4.2)	5 (10.4)	4 (8.3)	2 (4.2)	11 (22.9)	0.025
ALA	3 (1.3)	1 (2.1)	2 (4.2)	0	0	0	0.514
Vitamin D	70 (29.2)	14 (29.2)	16 (33.3)	19 (39.6)	12 (25.0)	9 (18.8)	0.349

ALA, Alpha-linolenic acid; BMI, body-mass index; EPA, eicosapentaenoic acid; DHA, docosahexaenoic acid; HRT, hormone replacement therapy; MET, metabolic equivalent of task minutes; OHA, oral hypoglycaemic agent; N-3 PUFA, omega-3 poly-unsaturated fat. Data reported as means ± SD for continuous variables and counts and (percentages) for categorical variables. Continuous data were compared using AVOVA or Kruskal–Wallis tests, and categorical data were compared using Fisher’s exact test. ^1^ European includes Northwest & Southeast descents. Other races include mixed heritage, North African and Middle Eastern, Peoples of the Americas, Australian Aboriginal and/or Torres Strait Islander. ^2^ National Health and Medical Research Council alcohol status and Dietitians Australia recommendations [27,28]. The reported >4 standard drinks at least once per month was within the time period of the last 3 months. ^3^ Participant currently taking medication/supplement as per medical history questionnaire. ^4^ In women only. ^5^ N-3 PUFA supplements were defined as EPA/DHA (fish- and krill-based), ALA (algae- and flaxseed-based). ^a,b,c^ Values within the same row without common superscript letters are significantly different (*p* < 0.05).

**Table 3:** Cardiometabolic disease risk factors of participants across different dietary pattern groups.

Variables (mmol/L)	Total Sample (*n* = 240)	Vegan (*n* = 48)	Lacto-Ovo Vegetarian (*n* = 48)	Pesco-Vegetarian (*n* = 48)	Semi-Vegetarian (*n* = 48)	Regular Meat-Eater (*n* = 48)	*p*
Hypertension ^1^	11 (4.6%)	0	3 (6.3%)	3 (6.3%)	1 (2.1%)	4 (8.3%)	0.261
T2D ^1^	2 (0.8%)	0	0	0	1 (2.1%)	1 (2.1%)	1.000
Hyperlipidaemia ^1^	9 (3.8%)	1 (2.1%)	2 (4.2%)	2 (4.2%)	2 (4.2%)	1 (4.2%)	0.910
SBP (mm Hg)	116.0 ± 14.7	114.6 ± 12.3	116.5 ± 14.6	114.5 ± 15.4	115.3 ± 12.2	119.0 ± 19.0	0.576
DBP (mm Hg)	72.2 ± 9.5	71.1 ± 9.6	72.4 ± 8.6	71.0 ± 9.1	72.5 ± 8.6	74.1 ± 11.3	0.534
Total cholesterol	5.2 ± 1.0	4.6 ± 0.9 ^a^	5.4 ± 1.1 ^b^	5.5 ± 1.0 ^b^	5.2 ± 0.7 ^b^	5.5 ± 0.8 ^b^	<0.001
LDL-C	3.2 ± 0.8	2.7 ± 0.7 ^a^	3.3 ± 0.9 ^b^	3.4 ± 0.8 ^b^	3.1 ± 0.7 ^a,b^	3.4 ± 0.7 ^b^	<0.001
HDL-C	1.6 ± 0.4	1.4 ± 0.3	1.6 ± 0.4	1.7 ± 0.4	1.6 ± 0.4	1.6 ± 0.4	0.070
Non-HDL-C	3.7 ± 0.9	3.2 ± 0.8 ^a^	3.8 ± 1.0 ^b^	3.8 ± 0.9 ^b^	3.6 ± 0.8 ^a,b^	3.9 ± 0.8 ^b^	<0.001
TG	1.1 ± 0.6	1.1 ± 0.5	1.2 ± 0.6	1.0 ± 0.1	1.1 ± 0.6	1.1 ± 0.5	0.439
FBG	4.7 ± 0.7	4.5 ± 0.5 ^a^	4.6 ± 0.6 ^a,b^	4.8 ± 0.6 ^a,b^	4.8 ± 1.0 ^a,b^	4.9 ± 0.8 ^b^	0.034
LFTs (U/L) ^2^ ALT AST GGT	23.8 ± 12.7 26.1 ± 11.7 19.8 ± 15.1	25 ± 13.0 26.9 ± 7.9 19.3 ± 20.5	25.3 ± 20.6 28.2 ± 23.1 19.6 ± 13.0	22.5 ± 7.8 24.7 ± 5.4 18.2 ± 8.4	21.6 ± 7.3 24.6 ± 5.4 18.0 ± 7.8	24.8 ± 9.8 26.1 ± 5.9 23.8 ± 20.3	0.493 0.487 0.325

ALT, alanine transaminase; AST, aspartate aminotransferase; FBG, fasting blood glucose; DBP, diastolic blood pressure; HDL-C, high-density lipoprotein cholesterol; LDL-C, low-density lipoprotein cholesterol; LFTs, liver function tests; OHA, oral hypoglycaemic agent; SBP, systolic blood pressure; T2D, type two diabetes; TG, triglycerides. Data reported as means ± SD for continuous variables and counts and (percentages) for categorical variables. Continuous data were compared using AVOVA or Kruskal–Wallis tests, and categorical data were compared using Fisher’s exact test. All values are in mmol/L unless otherwise specified. ^1^ The participant has been diagnosed and currently receiving treatment for the chronic disease as per their medical history questionnaire. ^2^ Data presented for *n* = 47 individuals in regular meat-eating dietary pattern group for LFTs. ^a,b^ Values within the same row without common superscript letters are significantly different (*p* < 0.05).

**Table 4:** Quantitative and qualitative dietary intake across dietary pattern groups derived from an average of two dietitian-administered diet histories and the AES^®^ FFQ.

Nutrient/Food Component (per/Day)	Total Sample (*n* = 240)	Vegan (*n* = 48)	Lacto-ovo Vegetarian (*n* = 48)	Pesco-Vegetarian (*n* = 48)	Semi-Vegetarian (*n* = 48)	Regular Meat-Eater (*n* = 48)	*p*
Quantitative data							
Energy (kJ)	9957 ± 2712	9784 ± 3254	9709.3 ± 3325	9013 ± 2284	9631 ± 2501	9491 ± 2304	0.506
Protein (%) ^1^	16.4% ± 4.1	15.4% ± 3.6 ^a,b^	14.8% ± 4.2 ^a^	16.8% ± 3.2 ^b^	15.4% ± 2.8 ^a,b^	19.8% ± 4.5 ^c^	<0.001
Carbohydrate (%) ^1^	40.2% ± 9.4	43.9% ± 8.4 ^a^	41.3% ± 8.9 ^a,b^	37.9% ± 8.9 ^b,c^	42.3% ± 8.7 ^a,b^	35.8% ± 9.9 ^c^	<0.001
Total fat (%) ^1^	37.3% ± 8.1	35.2% ± 8.4	38.2% ± 8.4	38.6% ± 7.8	36.5% ± 7.1	33.2% ± 14.6	0.227
Saturated (g)	28.6 ± 13.2	23.3 ± 11.2 ^a^	28.4 ± 13.9 ^a,b^	27.8 ± 13.3 ^a,b^	30.4 ± 11.1 ^a,b^	33.2 ± 14.6 ^b^	0.003
Trans fats (g)	1.0 ± 0.6	0.5 ± 0.7 ^a^	0.8 ± 0.5 ^a^	1.0 ± 0.6 ^b^	1.0 ± 0.6 ^b^	1.2 ± 0.6 ^b^	<0.001
MUFAs (g)	38.7 ± 15.1	37.0 ± 15.6	40.1 ± 15.8	39.9 ± 16.1	37.7 ± 14.6	39.1 ± 13.9	0.835
PUFAs (g)	20.1 ± 10.3	25.8 ± 13.6 ^a^	21.2 ± 10.0 ^a,b^	18.3 ± 7.4 ^b^	18.8 ± 9.8 ^b^	15.8 ± 6.8 ^b^	<0.001
Cholesterol (mg)	143.1 ± 136.5	33.6 ± 92.1 ^a^	98.2 ± 120.8 ^a,c^	157.9 ± 108.2 ^b^	155.7 ± 110.6 ^b,c^	270.2 ± 128.6 ^d^	<0.001
Dietary fibre (g)	45.3 ± 20.8	58.0 ± 18.8 ^a^	50.4 ± 29.7 ^a^	41.5 ± 13.6 ^b,c^	44.3 ± 16.9 ^b^	32.5 ± 11.0 ^c^	<0.001
Alcohol (g)	5.2 ± 10.1	1.8 ± 4.3 ^a^	3.5 ± 7.1 ^a^	7.2 ± 12.0 ^a,b^	4.1 ± 7.8 ^a,b^	9.4 ± 14.4 ^b^	0.001
Qualitative data							
Vegetables	5.9 ± 2.4	6.3 ± 2.5	6.2 ± 2.6	5.9 ± 2.1	5.9 ± 2.1	5.0 ± 2.6	0.081
Grains	2.7 ± 1.3	2.7 ± 1.1	2.6 ± 1.3	2.8 ± 1.5	2.7 ± 1.3	2.6 ± 1.5	0.907
Fruit	3.1 ± 1.7	3.8 ± 2.1 ^a^	3.0 ± 1.3 ^a,b^	3.0 ± 1.6 ^a,b^	3.1 ± 1.5 ^a,b^	2.7 ± 1.6 ^b^	0.015
Protein-rich foods ^2^	1.9 ± 0.9	1.5 ± 0.7 ^a^	1.3 ± 0.5 ^a^	2.0 ± 0.6 ^b^	2.0 ± 1.0 ^a,b^	2.7 ± 0.7 ^c^	<0.001
Meats/seafood ^2^	0.6 ± 0.8	0.0 ± 0.0 ^a^	0.0 ± 0.0 ^a^	0.6 ± 0.4 ^b^	0.6 ± 0.7 ^a,d^	1.7 ± 0.6 ^c^	<0.001
Legumes/nuts	1.1 ± 0.6	1.5 ± 0.7 ^a^	1.1 ± 0.5 ^b^	1.1 ± 0.5 ^b^	1.0 ± 0.5 ^b^	0.6 ± 0.5 ^c^	<0.001
Dairy ^2^	1.6 ± 1.3	0.0 ± 0.0 ^a^	1.6 ± 1.5 ^b^	1.8 ± 1.0 ^b^	1.7 ± 1.3 ^b^	2.0 ± 1.4 ^b^	<0.001
Discretionary ^3^ choices	1.7 ± 1.2	1.3 ± 1.0 ^a^	1.6 ± 1.1 ^a^	1.6 ± 1.1 ^a^	1.8 ± 1.2 ^a,b^	2.3 ± 1.5 ^b^	0.002

AES^®^, Australian Eating Survey; ARFS, Australian Recommended Food Score; FFQ, Food frequency questionnaire; MUFAs, monounsaturated fatty acids; PUFAs, polyunsaturated fatty acids. Quantitative dietary intake data are presented as an average of two dietitian-administered diet histories. Qualitative dietary intake data are presented from the AES FFQ. Data are reported as means ± SD or median (IQR) as appropriate. Data were assessed using ANOVA or Kruskal–Wallis tests. ^1^ Data for protein, carbohydrate and total fat are presented as % contribution of energy. ^2^ Protein-rich foods include meats/poultry/seafood/eggs/legumes/nuts. Assessment of meat exclusion among vegans and LOVs and dairy exclusion among vegans were derived from diet histories. ^3^ Included sugar sweetened beverages. ^a,b,c,d^ Values within a row without common superscript letters are significantly different (*p* < 0.05).

**Table 5:** Crude and adjusted associations of the 5-year and 10-year predicted risk of CVD between regular meat diets and plant-based diets.

Comparisons to RME	5-Year CVD Risk Score ^1^		10-Year CVD Risk Score ^2^	
Model	Crude	Adjusted	Crude	Adjusted
**Vegan**				
β	−2.40 *	−0.98	−3.20 *	−0.87
SE	0.88	0.58	1.20	0.57
95% CI	−4.12, −0.67	−2.11, 0.15	−5.55, −0.85	−1.20, 0.25
**Laco-ovo vegetarian diet**				
β	−0.67	−0.17	−0.95	−0.15
SE	0.93	0.68	1.31	0.68
95% CI	−2.48, 1.15	−1.50, 1.16	−3.51, 1.61	−1.48, 1.18
**Pesco-vegetarian diet**				
β	−0.65	−0.14	−0.47	0.37
SE	1.02	0.66	1.45	0.76
95% CI	−2.65, 1.36	−1.43, 1.14	−3.30, 2.37	−1.11, 1.85
**Semi-vegetarian diet**				
β	−1.25	−0.69	−1.55	−0.53
SE	0.91	0.56	1.27	0.72
95% CI	−3.05, 0.55	−1.79, 0.41	4.04, 0.94	−1.94, 0.88

Data are presented as β coefficients, standard errors and 95% CIs. Seemingly unrelated regression was performed to assess crude association between RMEs and PBDs and to adjust for confounding factors described in the Supplementary Figure S1, which include: physical activity, age, sex, smoking status and alcohol intake, with BMI assessed as a mediator. ^1^ 5-year risk calculated using the Framingham Risk Equation (25). ^2^ 10-year risk calculated using the Australian Absolute CVD Risk Calculator (26) * *p* < 0.05.

## Data Availability

Data described in the manuscript will be made available upon request from the corresponding author. The data are not publicly available due to privacy.

## Supplementary Materials

The following supporting information can be downloaded at: https://www.mdpi.com/article/10.3390/nu16071063/s1, Figure S1: Directed acyclic graph showing independence between potential confounding and mediating variables on the association between PBDs and CVD risk; Table S1: 5-year and 10-year predicted CVD risk scores by frequency of meat and fish intake per week across two categories within the meat- and fish-eating dietary patterns in the PBD Study; Table S2: 5-year and 10-year predicted CVD risk scores by frequency of meat and fish intake per week across three categories within the meat- and fish-eating dietary patterns in the PBD Study; Table S3: 5-year and 10-year predicted CVD risk score mean difference (compared to regular meat-eaters) by demographic subgroups across different plant-based diets in the PBD Study.

## References

[1] Roy Morgan Research The Slow but Steady Rise of Vegetarianism in Australia: Roy Morgan; 2016. https://www.roymorgan.com/findings/the-slow-but-steady-rise-of-vegetarianism-in-australia.

[2] Raven P. How Many Britons will Attempt a Vegan Diet and Lifestyle in January 2023?: YouGov UK; 2023. https://yougov.co.uk/topics/society/articles-reports/2022/12/29/how-many-britons-will-attempt-vegan-diet-and-lifes.

[3] Melina V., Craig W., Levin S. (2016). Position of the Academy of Nutrition and Dietetics: Vegetarian Diets. J. Acad. Nutr. Diet..

[4] Austin G., Ferguson J.J.A., Garg M.L. (2021). Effects of Plant-Based Diets on Weight Status in Type 2 Diabetes: A Systematic Review and Meta-Analysis of Randomised Controlled Trials. Nutrients.

[5] Huang R.Y., Huang C.C., Hu F.B., Chavarro J.E. (2016). Vegetarian Diets and Weight Reduction: A Meta-Analysis of Randomized Controlled Trials. J. Gen. Int. Med..

[6] Baleato C.L., Ferguson J.J.A., Oldmeadow C., Mishra G.D., Garg M.L. (2022). Plant-Based Dietary Patterns versus Meat Consumption and Prevalence of Impaired Glucose Intolerance and Diabetes Mellitus: A Cross-Sectional Study in Australian Women. Nutrients.

[7] Corrin T., Papadopoulos A. (2017). Understanding the attitudes and perceptions of vegetarian and plant-based diets to shape future health promotion programs. Appetite.

[8] Lea E., Worsley A. (2003). Benefits and barriers to the consumption of a vegetarian diet in Australia. Public Health Nutr..

[9] Kim H., Caulfield L.E., Garcia-Larsen V., Steffen L.M., Coresh J., Rebholz C.M. (2019). Plant-Based Diets Are Associated with a Lower Risk of Incident Cardiovascular Disease, Cardiovascular Disease Mortality, and All-Cause Mortality in a General Population of Middle-Aged Adults. J. Am. Heart Assoc..

[10] Casas R., Castro-Barquero S., Estruch R., Sacanella E. (2018). Nutrition and Cardiovascular Health. Int. J. Mol. Sci..

[11] Choi Y., Larson N., Steffen L.M., Schreiner P.J., Gallaher D.D., Duprez D.A., Shikany J.M., Rana J.S., Jacobs D.R. (2021). Plant-Centered Diet and Risk of Incident Cardiovascular Disease During Young to Middle Adulthood. J. Am. Heart Assoc..

[12] Jafari S., Hezaveh E., Jalilpiran Y., Jayedi A., Wong A., Safaiyan A., Barzegar A. (2022). Plant-based diets and risk of disease mortality: A systematic review and meta-analysis of cohort studies. Crit. Rev. Food Sci. Nutr..

[13] Mihrshahi S., Ding D., Gale J., Allman-Farinelli M., Banks E., Bauman A.E. (2017). Vegetarian diet and all-cause mortality: Evidence from a large population-based Australian cohort-the 45 and Up Study. Prev. Med..

[14] Tong T.Y.N., Appleby P.N., Bradbury K.E., Perez-Cornago A., Travis R.C., Clarke R., Key T.J. (2019). Risks of ischaemic heart disease and stroke in meat eaters, fish eaters, and vegetarians over 18 years of follow-up: Results from the prospective EPIC-Oxford study. BMJ.

[15] U.S. Department of Agriculture, U.S. Department of Health and Human Services Dietary Guidelines for Americans 2020–2025. https://www.dietaryguidelines.gov/sites/default/files/2020-12/Dietary_Guidelines_for_Americans_2020-2025.pdf.

[16] National Health and Medical Research Council (2013). Australian Dietary Guidelines.

[17] Ferguson J.J.A., Oldmeadow C., Mishra G.D., Garg M.L. (2022). Plant-based dietary patterns are associated with lower body weight, BMI and waist circumference in older Australian women. Public Health Nutr..

[18] Nutrition, Physical Activity & Obesity (NAO), Office for Prevention & Control of NCDs (MOS) (2021). Plant-based Diets and Their Impact on Health, Sustainability and the Environment: A Review of the Evidence.

[19] Ferguson J.J.A., Austin G., Oldmeadow C., Garg M.L. (2023). Plant-Based Dietary Patterns and Cardiovascular Disease Risk in Australians: Protocol for a Cross-Sectional Study. Nutrients.

[20] EatForHealth Australian Guide to Healthy Eating: Australian Goverment National Health and Medical Researcg Council. 2013. https://www.eatforhealth.gov.au/guidelines/australian-guide-healthy-eating.

[21] Collins C.E., Boggess M.M., Watson J.F., Guest M., Duncanson K., Pezdirc K., Rollo M., Hutchesson M.J., Burrows T.L. (2014). Reproducibility and comparative validity of a food frequency questionnaire for Australian adults. Clin. Nutr..

[22] Shrier I., Platt R.W. (2008). Reducing bias through directed acyclic graphs. BMC Med. Res. Methodol..

[23] Australian Chronic Disease Prevention Alliance (ACDPA) Australian Absolute Cardiovascular Disease Risk Calculator 2012. https://www.cvdcheck.org.au/.

[24] National Vascular Disease Prevention Alliance (2012). Guidelines for the Management of Absolute Cardiovascular Disease Risk.

[25] D’Agostino R.B., Vasan R.S., Pencina M.J., Wolf P.A., Cobain M., Massaro J.M., Kannel W.B. (2008). General cardiovascular risk profile for use in primary care: The Framingham Heart Study. Circulation.

[26] Lu Y., Hajifathalian K., Ezzati M., Woodward M., Rimm E.B., Danaei G. (2014). Metabolic mediators of the effects of body-mass index, overweight, and obesity on coronary heart disease and stroke: A pooled analysis of 97 prospective cohorts with 1·8 million participants. Lancet.

[27] Australian Government National Health and Medical Research Council (2009). Australian Guidelines to Reduce Health Risks from Drinking Alcohol Canberra.

[28] Alcohol Guidlines: Dietitians Australia. 2013. https://www.eatforhealth.gov.au/.

[29] Craig C.L., Marshall A.L., Sjöström M., Bauman A.E., Booth M.L., Ainsworth B.E., Pratt M., Ekelund U., Yngve A., Sallis J.F. (2003). International Physical Activity Questionnaire: 12-Country Reliability and Validity. Med. Sci. Sports Exerc..

[30] Body Mass Index (BMI) and Waist Measurement: Australian Goverment, Department of Health and Aged Care. 2021. https://www.health.gov.au/topics/overweight-and-obesity/bmi-and-waist.

[31] So J.H., Lee J.K., Shin Jy Park W. (2016). Risk of Cardiovascular Disease Using Framingham Risk Score in Korean Cancer Survivors. Korean J. Fam. Med..

[32] Imai K., Keele L., Tingley D. (2010). A general approach to causal mediation analysis. Psychol. Methods.

[33] Dybvik J.S., Svendsen M., Aune D. (2023). Vegetarian and vegan diets and the risk of cardiovascular disease, ischemic heart disease and stroke: A systematic review and meta-analysis of prospective cohort studies. Eur. J. Nutr..

[34] Quek J., Lim G., Lim W.H., Ng C.H., So W.Z., Toh J., Pan X.H., Chin Y.H., Muthiah M.D., Chan S.P. (2021). The Association of Plant-Based Diet with Cardiovascular Disease and Mortality: A Meta-Analysis and Systematic Review of Prospect Cohort Studies. Front. Cardiovasc. Med..

[35] Gan Z.H., Cheong H.C., Tu Y.K., Kuo P.H. (2021). Association between Plant-Based Dietary Patterns and Risk of Cardiovascular Disease: A Systematic Review and Meta-Analysis of Prospective Cohort Studies. Nutrients.

[36] Dinu M., Abbate R., Gensini G.F., Casini A., Sofi F. (2017). Vegetarian, vegan diets and multiple health outcomes: A systematic review with meta-analysis of observational studies. Crit. Rev. Food Sci. Nutr..

[37] Huang T., Yang B., Zheng J., Li G., Wahlqvist M.L., Li D. (2012). Cardiovascular disease mortality and cancer incidence in vegetarians: A meta-analysis and systematic review. Ann. Nutr. Metab..

[38] Petrea R.E., Beiser A.S., Seshadri S., Kelly-Hayes M., Kase C.S., Wolf P.A. (2009). Gender differences in stroke incidence and poststroke disability in the Framingham heart study. Stroke.

[39] Gallucci G., Tartarone A., Lerose R., Lalinga A.V., Capobianco A.M. (2020). Cardiovascular risk of smoking and benefits of smoking cessation. J. Thorac. Dis..

[40] Threapleton D.E., Greenwood D.C., Evans C.E.L., Cleghorn C.L., Nykjaer C., Woodhead C., Cade J.E., Gale C.P., Burley V.J. (2013). Dietary fibre intake and risk of cardiovascular disease: Systematic review and meta-analysis. BMJ Br. Med. J..

[41] Australian Government NHaMRC Nutrient Reference Values: Australian Goverment 2006. https://www.eatforhealth.gov.au/nutrient-reference-values/nutrients/.

[42] de Souza R.J., Mente A., Maroleanu A., Cozma A.I., Ha V., Kishibe T., Uleryk E., Schunemann H., Beyene J., Anand S.S. (2015). Intake of saturated and trans unsaturated fatty acids and risk of all cause mortality, cardiovascular disease, and type 2 diabetes: Systematic review and meta-analysis of observational studies. BMJ.

[43] Hooper L., Martin N., Jimoh O.F., Kirk C., Foster E., Abdelhamid A.S. (2020). Reduction in saturated fat intake for cardiovascular disease. Cochrane Database Syst. Rev..

[44] Guasch-Ferré M., Babio N., Martínez-González M.A., Corella D., Ros E., Martín-Peláez S., Estruch R., Aros F., Gomez-Garcia E., Fiol M. (2015). Dietary fat intake and risk of cardiovascular disease and all-cause mortality in a population at high risk of cardiovascular disease. Am. J. Clin. Nutr..

[45] Ander B.P., Dupasquier C.M., Prociuk M.A., Pierce G.N. (2003). Polyunsaturated fatty acids and their effects on cardiovascular disease. Exp. Clin. Cardiol..

[46] Ferguson J.J.A., Dias C.B., Garg M.L., Hegde M.V., Zanwar A.A., Adekar S.P. (2016). Omega-3 Polyunsaturated Fatty Acids and Hyperlipidaemias. Omega-3 Fatty Acids: Keys to Nutritional Health.

[47] Wang T., Kroeger C.M., Cassidy S., Mitra S., Ribeiro R.V., Jose S., Masedunskas A., Senior A.M., Fontana L. (2023). Vegetarian Dietary Patterns and Cardiometabolic Risk in People with or at High Risk of Cardiovascular Disease: A Systematic Review and Meta-analysis. JAMA Netw. Open.

[48] Koch C.A., Kjeldsen E.W., Frikke-Schmidt R. (2023). Vegetarian or vegan diets and blood lipids: A meta-analysis of randomized trials. Eur. Heart J..

[49] Yokoyama Y., Barnard N.D., Levin S.M., Watanabe M. (2014). Vegetarian diets and glycemic control in diabetes: A systematic review and meta-analysis. Cardiovasc. Diagn. Ther..

[50] McMacken M., Shah S. (2017). A plant-based diet for the prevention and treatment of type 2 diabetes. J. Geriatr. Cardiol..

[51] Shi W., Huang X., Schooling C.M., Zhao J.V. (2023). Red meat consumption, cardiovascular diseases, and diabetes: A systematic review and meta-analysis. Eur. Heart J..

[52] Foundation H. Meat & Heart Healthy Eatin: Dietary Position Statement: The Australian Heart Foundation; 2019. www.heartfoundation.org.au.

[53] Nohr E.A., Liew Z. (2018). How to investigate and adjust for selection bias in cohort studies. Acta Obstet. Gynecol. Scand..

