# Supplementary material for: Cardiovascular Disease Risk in Individuals Following Plant-Based Dietary Patterns Compared to Regular Meat-Eaters
Source: Nutrients. 2024 Apr 5;16(7):1063. doi: 10.3390/nu16071063 (PMC11013900; doi:10.3390/nu16071063)
Supplement: Supplementary file 1 [file nutrients-16-01063-s001.zip › nutrients-2913369-supplementary.pdf]

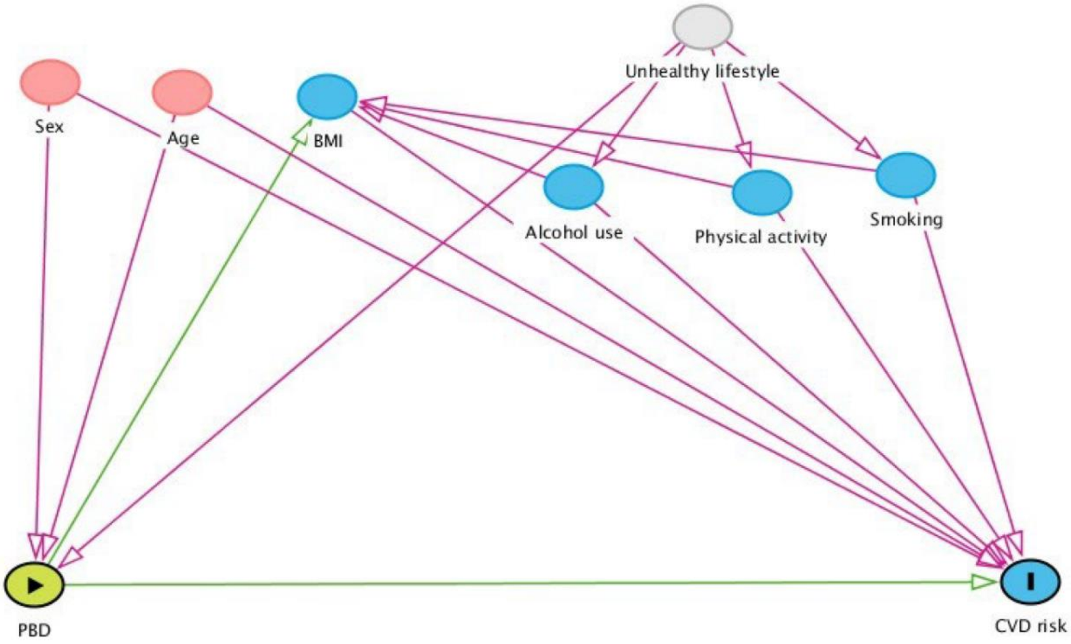

Supplementary Figure S1. Directed acyclic graph showing independence between potential confounding and mediating variables on the association between PBDs and CVD risk. Confounding factors are age, sex, alcohol use, smoking status, and physical activity levels and BMI is a mediator.BMI, body mass index; PBD, plant-based diets; CVD, cardiovascular disease.

Supplementary Table S1. 5-year and 10-year predicted CVD risk scores by of frequency of meat and fish intake per week across two categories within the meat and fish-eating dietary patterns in the PBD Study.

	≤3 times per week	>3 times per week	*p*	Adjusted *p*
**Meat & Fish**	*n* = 52	*n* = 92		
5-year CVD risk [1]	3.19 ± 3.87	3.57 ± 4.36	0.608	0.624
10-year CVD risk [1]	6.56 ± 6.87	6.83 ± 6.24	0.811	0.284
**Fish**	*n* = 98	*n* = 46		
5-year CVD risk [1]	3.24 ± 3.95	3.83 ± 4.64	0.438	0.908
10-year CVD risk [1]	6.49 ± 6.3	7.26 ± 6.81	0.505	0.732
**Red meat**	*n* = 95	*n* = 49		
5-year CVD risk [1]	2.92 ± 3.27	4.41 ± 5.44	0.043	0.107
10-year CVD risk [1]	6.34 ± 6.02	7.51 ± 7.23	0.305	0.798

CVD, cardiovascular disease. Data reported as means ± SD or median (IQR) as appropriate for distribution. Seemingly unrelated regression was preformed to crude and adjust outcomes with confounding factors described in the Supplementary Figure 1 which include: physical activity, age, sex, smoking status, alcohol intake, with BMI as assessed as a mediator; [1] 5-year risk calculated using the Framingham Risk Equation (25) and 10-year risk calculated using the Australian Absolute CVD Risk Calculator (26).

Supplementary Table S2. 5-year and 10-year predicted CVD risk scores by frequency of meat and fish intake per week across three categories within the meat and fish-eating dietary patterns in the PBD Study.

	≤2 times per week	>2 to <7 times per week	Daily or multiple times a day	*p*
**Meat & Fish**	(*n* = 31)	(*n* = 54)	(*n* = 59)	
5-year CVD risk [1]	3.06 ± 3.72	3.09 ± 3.22	3.92 ± 5.09	0.834
10-year CVD risk [1]	5.99 ± 6.07	6.61 ± 6.29	7.24 ± 6.85	0.560
**Fish**	(*n* = 83)	(*n* = 53)	(*n* = 8)	
5-year CVD risk [1]	3.1 ± 3.96	4.21 ± 4.65	1.75 ± 1.8	0.161
10-year CVD risk [1]	6.01 ± 5.7	8.07 ± 7.54	5.41 ± 5.16	0.160
**Red meat**	(*n* = 81)	(*n* = 43)	(*n* = 20)	

5-year CVD risk [1]	2.86 ± 3.29	3.67 ± 3.96	5.2 ± 6.8	0.073
10-year CVD risk[1]	6.21 ± 6.18	6.78 ± 5.31	8.78 ± 9.21	0.282

CVD, cardiovascular disease. Data reported as means ± SD or median (IQR) as appropriate for distribution. [1] 5-year risk calculated using the Framingham Risk Equation (25) and 10-year risk calculated using the Australian Absolute CVD Risk Calculator (26).

Supplementary Table S3. 5-year and 10-year predicted CVD risk score mean difference (compared to regular meat-eaters) by demographic subgroups across different plant-based diets in the PBD Study.

**Comparisons to Regular Meat-eaters**	**5-year Risk Score [1]**		**10-year Risk Score [1]**	
**Length of dietary**	**≤10 year (*n* = 138)**	**>10 year (102)**	**≤10 year (138)**	**>10 year (102)**
Vegan	-1.22 (-2.94, 0.49)	-1.41 (-4.21, 1.38)	-2.6 (-5.01, -0.01)*	-0.35 (-3.87, 3.17)
Lacto-ovo vegetarian	-1.15 (-2.89, 0.582)	0.9 (-1.03, 2.84)	-2.42 (-5.02, 0.17)	1.36 (-0.22, 2.95)
Pesco-vegetarians	0.45 (1.87, 2.77)	-0.18 (-1.83, 1.46)	-0.73 (-3.84, 2.38)	0.92 (-0.81, 2.66)
Semi-vegetarians	-0.96 (-2.77, 0.84)	0.11 (-1.52, 1.74)	-2.19 (4.84, 0.45)	0.76 (-1.11, 2.64)
p-value [2]	*p* = <0.001	*p* = <0.001	-	-
**Age**	**≤60 years (*n* = 163)**	**>60 years (*n* = 77)**	**≤60 years (*n* = 163)**	**>60 years (*n* = 77)**
Vegan	-0.95 (-3.79, 1.88)	-2.42 (-5.74, 0.91)	-0.42 (1.55, 0.71)	-4.42 (-9.06, 0.23)
Lacto-ovo vegetarian	-0.38 (-3.29, 2.53)	-0.22 (-3.221, 2.78)	0.75 (-1.01, 1.21)	-0.45 (-3.2, 2.29)
Pesco-vegetarians	-1.29 (-4.02, 1.24)	-0.52 (-3.03, 1.99)	0.33 (-0.91, 1.57)	0.36 (-2.22, 2.95)
Semi-vegetarians	-0.58 (-3.33, 2.16)	-1.85 (-3.94, 0.24)	0.8 (-0.53, 2.19)	-1.65 (-4.01, 0.8)
p-value [2]	*p* = <0.001	*p* = <0.001	-	-
**BMI[3]**	**Not overweight or obese (*n* = 143)**	**Overweight or obese (*n* = 97)**	**Not overweight or obese (*n* = 143)**	**Overweight or obese (*n* = 97)**
Vegan	-0.45 (-1.71, 0.81)	-1.06 (-3.13, 1.02)	-0.37 (-1.6, 0.85)	-0.13 (-2.02, 1.94)
Lacto-ovo vegetarian	0.95 (-0.85, 2.76)	-0.64 (-2.37, 1.08)	045 (-1.13, 2.04)	0.43 (-1.75, 2.62)
Pesco-vegetarians	0.25 (-1.39, 1.81)	-0.23 (-2.36, 1.9)	0.42 (-0.87, 1.71)	0.79 (-1.7, 3.28)
Semi-vegetarians	-0.46 (-1.71, 0.78)	-0.57 (-2.32, 1.17)	-0.73 (-1.99, 0.54)	0.55 (-1.98, 2.07)
p-value [2]	*p* = <0.001	*p* = <0.001	-	-
**Sex**	**Men (*n* = 54)**	**Women (*n* = 186)**	**Men (*n* = 54)**	**Women (*n* = 186)**
Vegan	-2.67 (-5.68, 0.34)	0.31 (-1.98, 2.59)	-3.12 (-6.37, 0.15)	0.13 (-0.79, 1.05)
Lacto-ovo vegetarian	-2.16 (-5.41, 1.09)	0.58 (-1.62, 2.79)	-2.41 (-6.39, 1.57)	0.58 (-.048, 1.64)
Pesco-vegetarians	-0.59 (-3.49, 2.3)	0.1 (-2.02, 2.22)	1.28 (-3.45, 6.01)	0.26 (-0.61, 1.12)
Semi-vegetarians	-2.42 (-5.54, 0.69)	0.00 (-2.18, 2.17)	-3.74 (-7.83, 0.35)	0.31, (-0.79, 1.41)
p-value [2]	*p* < 0.001	*p* = <0.001	-	-
**Smoking status**	**No (*n* = 225)**	**Yes (*n* = 15)**	**No (*n* = 225)**	**Yes (*n* = 15)**
Vegan	-0.8 (-1.8, 1.96)	0.62 (-17.48, 18.7)	-0.61 (-1.76, 0.55)	1.77 (-24.67, 28.2)
Lacto-ovo vegetarian	0.13 (-1.15, 1.41)	-0.92 (-17.89, 16.06)	0.12 (-1.32, 1.56)	0.3 (-24.5, 25.1)
Pesco-vegetarians	0.13 (-0.94, 1.21)	1.86 (-20.64, 24.36)	0.55 (-0.75, 1.85)	3.5 (-29.31, 36.4)
Semi-vegetarians	-0.65 (-1.58, 0.27)	2.09 (-13.78, 17.96)	-0.58 (-1.91, 0.74)	6.09 (-17.09, 29.27)
p-value [2]	*p* = <0.001	*p* = 0.277	-	-
**Treatment of chronic diseases**	**No (*n* = 220)**	**Yes (*n* = 20)**	**No (*n* = 220)**	**Yes (*n* = 20)**
Vegan	-0.54 (-1.61, 0.54)	-4.42 (-18.67, 9.82)	-0.53 (1.72, 0.65)	5.65 (-13.88, 15.18)
Lacto-ovo vegetarian	0.34 (-1.02, 1.7)	-9.52 (-24.07, 5.04)	0.24 (-1.16, 1.64)	-10.35 (-50.598, 29.9)
Pesco-vegetarians	0.44 (-0.79, 1.67)	-4.14 (-14.03, 5.75)	0.37 (-1.03, 1.77)	2.66 (-15.01, 20.37)
Semi-vegetarians	-0.07 (-0.98, 0.85)	-5.29 (-17.62, 6.84)	-0.07 (-1.43, 1.29)	0.464 (-27.95, 28.8)
p-value [2]	*p* = <0.001	*p* = <0.001	-	-

BMI, body mass index; CVD, cardiovascular disease. Data presented as predicted CVD risk scores means (95% CI). Seemingly unrelated regression was preformed to adjust for confounding factors described in the Supplementary Figure 1 which include: physical activity, age, sex, smoking status, alcohol intake, with BMI assessed as a mediator. [1] 5-year risk calculated using the Framingham Risk Equation (25) and 10-year risk calculated using the Australian Absolute CVD Risk Calculator (26). [2] Chi-square goodness of fit score associated *p*-value from seemingly unrelated multiple regression. [3] Overweight or obese defined as a body-mass index of ≥25kg/m^2 as per WHO definitions (31). * p <0.05.
